# Kinetics Evaluation of IgM and IgG Levels in the Mice Infected with *Trichinella spiralis* Experimentally Using ES Antigens from Different Developmental Stages of the Parasite

**Published:** 2019

**Authors:** Cheng-Cheng ZHAI, Zhao-Jin SUN, Ming-Yuan LIU, Xiao-Lei LIU, Xue BAI, Xue-Lin WANG, Xiu-Ping WU, Jia-Xu CHEN

**Affiliations:** 1. National Institute of Parasitic Diseases, Chinese Center for Disease Control and Prevention, WHO Collaborating Center for Tropical Diseases, Key Laboratory of Parasite and Vector Biology, National Health and Family Planning Commission, Shanghai 200025, China; 2. Key Laboratory for Zoonoses Research, Ministry of Education, Institute of Zoonoses, Jilin University, Changchun, China

**Keywords:** *Trichinella spiralis*, ES antigen, IgG, IgM, ELISA

## Abstract

**Background:**

To assay the *Trichinella*-specific IgM and IgG antibody responses during the early stage of infection, serum was collected from mice infected with the muscle larvae (ML) of *T. spiralis* (ISS534) at different dpi (days post infection) up to 60 days.

**Methods:**

The levels of IgM and IgG antibodies in serum were measured by ES antigens from different stage of *T. spiralis* using the ELISA method in Shanghai, China in 2017.

**Results:**

The anti-*Trichinella* IgM and IgG could be detected by ES antigens from the adult three days worm (Ad3) as early as 5 dpi and 8 dpi, respectively. ES antigens from the mixture of adult six days worm & new born larvae (Ad6+NBL) was similar to Ad3. When antibodies were detected by these two antigens, the levels of IgM peaked at 14 dpi and then declined from 15 dpi to 60 dpi; the IgG peaked at 20 dpi, and gradually declined, however, higher detection levels were maintained until 60 dpi.

**Conclusion:**

Ad3 ES antigens showed more antigenicity than Ad6+NBL ES on titer detection of IgM and IgG antibodies, and the production of Ad3 ES is easier. In terms of early diagnosis, these two antigens are better than the ML ES antigens of *T. spiralis*, which antibodies could not be detected before 20dpi. Ad3 ES antigens might be good candidate for the early diagnosis of trichinellosis or the mixture of Ad3 and Ad6+NBL ES might be used.

## Introduction

*T*richinellosis is caused by nematodes of the genus *Trichinella*, a widespread meat-borne zoonotic nematode genus that infects a broad range of animal species (1). This disease has an important impact worldwide, not only on socio-economic factors such as the international trade of animals but also as a public health problem (2). The International Commission on Trichinellosis (ICT) reported total about 65818 cases of human trichinellosis from 1986 to 2009 (3). However, the early clinical diagnosis of human trichinellosis is rather difficult because pathological signs or symptoms are lacking (4). A variety of serological assays have been described for the diagnosis of trichinellosis, such as ELISA, which is the only method endorsed by the ICT (5). However, it is not available for the detection of early-stage *Trichinella* infection in individual animal due to the lack of proper antigens.

*T. spiralis* infection is initiated by the consumption of meat contaminated with infective muscle larvae (ML). After infection, ML develops into adult worms (Ad) in the small intestine, and the female worms start to release newborn larvae (NBL) at 5 d post infection (dpi). The NBL migrate through the lymphatic and blood vessels, invade striated muscle cells, and develop into the infective Ll stage over a period of 2–3 wk, capable of infecting the next host, thus completing the life cycle (6). The persistent release of excretory/secretory (ES) antigens by *T. spiralis* may play a major role in sustaining the host response, and the ES antigens secreted by the ML stage of *T. spiralis* are the most commonly used diagnostic antigens for trichinellosis, as recommended by the ICT (7, 8). However, the ML ES antigens could not recognize early antibodies induced by Ad and NBL, there is a dangerous “diagnostic blind spot” in which early *T. spiralis* infection cannot be detected, while the NBL can develop into infectious larvae at 18 dpi (9). Thus, ML ES antigens could be used to detect sera in late infection of *Trichinella*. More importantly, ML ES antigens cross-react with the sera of patients with other types of helminthiasis (10, 11). Therefore, it is necessary to screen sensitive and specific early diagnostic antigens of *Trichinella*.

*T. spiralis* exhibits different antigen expression at different developmental stages (12). The adult and newborn larvae are present during the early stage of *Trichinella* infection. Their ES antigens are the first antigens the immune system exposed to during early infection and may induce the host to produce an antibody response. The earlier classes of specific antibodies (IgM/IgG) are bound to *T. spiralis* antigens and form immune complexes, so they are present in infected hosts at the beginning of the infection (13, 14). Moreover, the first line of antibodies produced in the humoral response belongs to the IgM class, expressed without isotype switching (15).

We evaluated the early diagnostic value of adult and newborn larval stages by analyzing the dynamic curves of *Trichinella*-specific IgM and IgG antibodies.

## Materials and Methods

### Parasite and ethics statement

*T. spiralis* (T1, ISS534 isolates) was maintained by serial passage in ICR Wistar rats in Institute of Zoonoses of Jilin University. Larvae were collected by artificial digestion, performed using a standard protocol (16).

The animals were treated in strict accordance with the National Institutes of Health guidelines (publication no. 85–23, revised 1996). The animal protocols were approved by the Ethical Committee of Jilin University, affiliated with the Provincial Animal Health Committee, Jilin Province, China (Ethical Clearance number IZ-2009-08).

### Experimental infection and serum samples

Forty 6-week-old specific pathogen-free (SPF) female BALB/c mice were randomly divided into two groups (twenty mice per group): an infected group (orally inoculated with 300 *T. spiralis* larvae) and a non-infected group as a negative control. Tail blood (100–150 μl) was collected at 0, 5, 8, 11, 14, 17, 20, 23, 26, 29, 32, 35, 40, 45, 50, 55 and 60 dpi in Shanghai, China in 2017. The serum was isolated from the samples by centrifugation and was subsequently stored at −20 °C until use (17).

### Preparation of ES antigens from different stages of T. spiralis

ES antigens from different stage of *T. spiralis* were prepared (18, 19). Briefly, muscle larvae (ML) was recovered by digestion from Wistar rats infected with 8000 larvae at 35 d post-infection (dpi). Adult worms were isolated from the small intestines of experimentally infected Wistar rats at 3 and 6 dpi (19). The isolated parasites were washed with stroke-physiological saline solution supplemented with 2% antibiotics (penicillin and streptomycin) at 37 °C and 5% atmospheric CO_2_ for 24 h, followed by washing three times in RPMI 1640 medium supplemented with double antibiotics. After they were washed, the parasites were incubated in the same medium with 1% antibiotic concentration at a density of 2000 worms/ml for 12 h at 37 °C in 5% CO_2_. After incubation, the media was poured into conical bottom pilsner glasses, and the parasites were allowed to settle for 10 min. The supernatant containing the ES antigens was filtered through a 0.22 μm membrane and concentrated in Ultra-15 3K centrifugal filters at 3000 rpm (20). ES antigen concentrations were determined by the Bradford assay and were stored at −20 °C until use.

### Analysis of anti-Trichinella IgM and IgG by ELISA

Serum samples were examined by ELISA for anti-*Trichinella* IgM and IgG antibodies using ES antigens from three different stages of *T. spiralis*. The optimal dilutions of various reagents were first determined by checkerboard titration. In brief, a 96-well ELISA plate (Costar, Cambridge, Massachusetts) was coated with different ES antigens (10 μg/ml, in 100 μl carbonate buffer, pH 9.6), incubated for 2 h at 37 °C, and moved to 4 °C overnight. Plates were washed three times (5 min each wash) in wash buffer (PBS supplemented with 0.05% Tween-20, PBST). Then, the plates were incubated with 200 μl blocking buffer (PBST supplement with 5% non-fat dry milk) in each well for 2 h and washed three times. Serum samples diluted at 1:50 in blocking buffer were added to the wells, and the plates were incubated for 1 h at 37 °C and then washed three times. Then, 100 μl of HRP-conjugated goat anti-mouse IgM and IgG (diluted to 1:3000 in blocking buffer, Sigma, USA) was added to the wells, and the plates were incubated for 1 h at 37 °C. After the final three items of washing, 100 μl of TMB substrate solution (Sigma) was added to the wells, and after 10-min incubation, 50 μl of stop buffer (2 M H_2_SO_4_) was added to each well. Optical density was read at 450 nm using an automated microplate reader (Vmax, Molecular Devices). The cut-off value of the ELISA was evaluated for the three antigens based on 2.1 times the average OD value of the negative samples (21).

### Statistical analysis

All results were expressed as the mean±SD. Statistical analysis was performed using the GraphPad Prism 6.0 software. One-way and two-way analysis of variance (ANOVA) were used to compare significant differences at antibody levels by different ES antigens. *P<0.05 was considered statistically significant.

## Results

### Kinetics of specific IgM antibodies with ES antigens from three different stages of T. spiralis

**Fig. 1:**
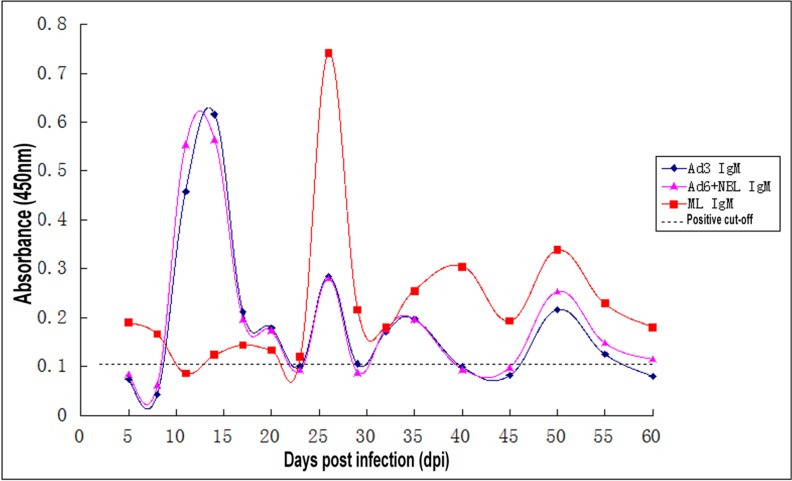
Kinetics of anti-*Trichinella* IgM antibodies in mice sera detected using ES antigens from three different stages of *T. spiralis*

The levels of anti-*Trichinella* IgM in the serum samples of the mice infected with 300 *T. spiralis* muscle larvae were determined by ELISA with the three development stages of ES antigens (Ad3, Ad6 + NBL, and ML). Anti-*Trichinella* IgM could first be detected at 5 dpi both by ES antigens of Ad3 and Ad6+NBL (Fig. 1). The levels of IgM antibodies peaked at 14 dpi and gradually declined until 60 dpi. The levels of anti-*Trichinella* IgM detected by ELISA with the Ad3 ES and Ad6+NBL ES antigens reappeared with another minor peak at 26 dpi, first observed by ML ES antigens, with the minor peak. For ML ES antigens, IgM antibody was maintained at a low level and could be detected until 60 dpi (Fig. 1). Serum anti-*Trichinella* IgM levels determined by ELISA with the three antigens were statistically significant (*P*<0.05).

### Kinetics of specific IgG antibodies with ES antigens from three different stages of T. spiralis

**Fig. 2:**
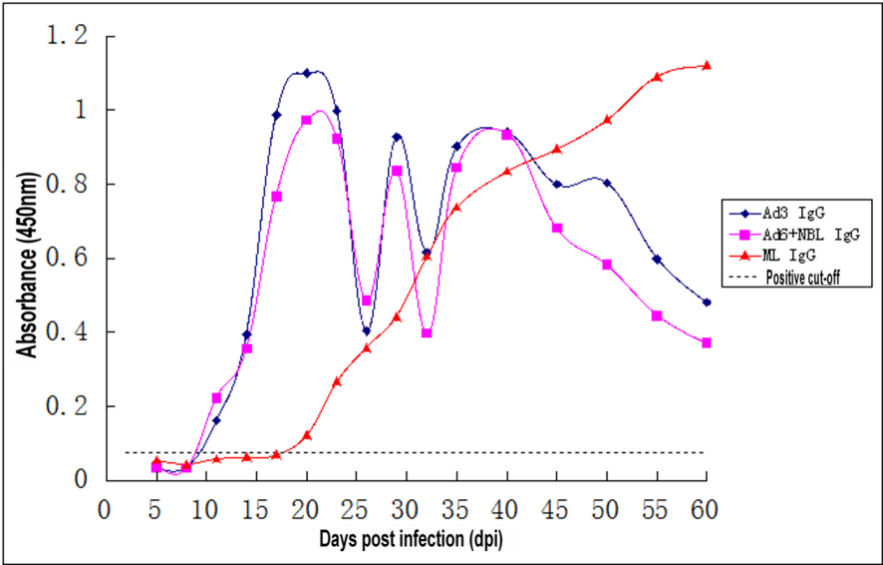
Kinetics of anti-*Trichinella* IgG antibodies in mice sera detected using ES antigens from three different stages of *T. spiralis*

Serum anti-*Trichinella* IgG antibodies in serum samples of mice infected with 300 *T. spiralis* muscle larvae were also assayed by ELISA with the three development stages of ES antigens (Ad3, Ad6 + NBL, and ML), and the results are shown in Fig. 2. For the two early antigens (Ad3, Ad6 + NBL), the anti-*Trichinella* IgG antibodies began to be produced at 8 dpi, peaked at 20 dpi, and gradually declined; however, higher detection levels were maintained until 60 dpi. Using ML ES antigens to detect serum IgG antibody levels showed that antibody responses could be detected at 20 dpi, following a constant increase throughout the remaining experimental time. Serum anti-*Trichinella* IgG levels determined by ELISA with the three antigens were statistically significant (*P*<0.05).

## Discussion

The clinical symptoms caused by *T. spiralis* infection are quite complex, and muscle biopsy sampling is not sufficiently sensitive to detect *Trichinella* larvae in light infections (20). Immunological methods for the diagnosis of trichinellosis that detect specific anti-*Trichinella* IgG antibodies by ML ES in serum are currently preferred (22). However, different antigens are expressed at different developmental stages of *T. spiralis*, and the ML ES antigens are not exposed to the host’s immune system until the NBL invade striated muscle cells and develop into the infective Ll stage over a period of 2–3 wk (23). In addition, it is different from ML ES, the Ad3 ES and Ad6+NBL ES antigens involve the earliest interaction between the host and parasite and might be better early diagnostic markers of trichinellosis. Thus, the presence or absence of IgG antibody might not be an appropriate and reliable indicator of infection in the early diagnosis of *Trichinella* infection because the serum IgG antibodies appear later in the immune response in the body (8, 14, 24). Serum IgM antibodies appear in the early stage of infection, having greater significance in the early diagnosis of *Trichinella* infection (25).

Mice infected with a dose of 400 larvae of *T. spiralis* exhibit an increase in the specific IgM antibody response until 20 dpi, as determined by an ELISA based on the ML ES antigen (8). Similar results were obtained in a study of mice with low-dose infection, demonstrating IgM antibody production at 30 dpi that was slightly above the cut-off (26). Therefore, in the present study, the serum levels of IgM and IgG were investigated by ELISA with ES antigens from three different stages of *T. spiralis*. Consistent with previous studies, our results demonstrated the specific anti-*Trichinella* IgM antibodies were not significantly detected until 26 dpi and then decreased rapidly, as detected by ML ES. Instead, specific-IgM antibodies were detectable by ES antigens of Ad3 and Ad6+NBL as early as 5 dpi, suggesting that the Ad3 ES and Ad6+NBL ES antigens could induce an earlier antibody response compared to ML ES antigens. IgM is the first isotype produced prior to class switching and can effectively recognize and eliminate pathogens in the early stage of immune defense (27, 28). An increased specific IgM antibody response is typical for acute infection, and then IgM levels drastically decline due to the short half-life (15). Some IgM is also produced in secondary and subsequent responses, although other isotypes (mainly IgG) dominate the later phases of the antibody response (29). This phenomenon may explain why the specific anti-*Trichinella* IgM antibodies were maintained at a low level until 60 dpi in this study. Intriguingly, the levels of anti-*Trichinella* IgM by ELISA with the Ad3 ES and Ad6+NBL ES antigens rebounded with another minor peak at 26 dpi, which the first detected with ML ES antigens, suggesting that the cross-reactive antigens existed in ES antigens from different stage of *T. spiralis.*

The specific anti-*Trichinella* IgG antibodies showed different characteristic in this experiment. The level of IgG detected by ES antigens of Ad3 and Ad6+NBL was higher than that detected by ML ES antigens before 26 dpi. Ad3 ES and Ad6+NBL ES antigens were more sensitive than the ML ES antigens for early serodiagnosis of trichinellosis. As the major serum isotype, IgG antibodies are generated following class switching and maturation of the antibody response and, thus, participate predominantly in the secondary immune response (30). Specific anti-*Trichinella* IgG antibodies can also be found at 6 or 8 months after infection and even 2 years after infection (14). The stimulation of ES antigen by larvae and their penetration through capsules and the degradation of larvae by inflammatory cells explain the long-lasting presence of anti-*Trichinella* IgM and IgG antibodies (30, 31). Similar study showed that in mice infected with 100ML, anti-*Trichinella* IgG was first detected by ELISA with the AW ES antigens, crude antigens, ML ES antigens 8,12 and 12 d post-infection (dpi), respectively. In mice infected with 500 ML, specific antibodies were first detected by ELISA with the three antigen preparations at 10, 8,10 dpi, respectively (32). In this study, although specific anti-*Trichinella* IgG antibodies were detected until 20 dpi by ML ES antigens, the IgG levels were significantly elevated and remained high from 26 to 60 dpi, indicating that ML ES antigens have advantages for the late diagnosis of trichinellosis.

Different hosts may have different immune responses when fighting with the infection of *Trichinella*. However, in this study, mice were only used as animal model, and the titre of IgM detected by the Ad3 ES antigens was higher before 14 dpi compared to the titre of IgG detected by ELISA with the Ad3 ES antigens. Instead, the titre of IgG detected by the Ad3 ES antigens was higher between 14 and 26 dpi than at other points. Thus, we conclude that the Ad3 ES antigens can be used for the detection IgM at the early serodiagnosis time points (before 14 dpi) and can be used for the detection IgG at the mid-late serodiagnosis time points (14–26 dpi).

## Conclusion

The sensitivity of Ad3 ES antigens and Ad6+NBL ES antigens of *T. spiralis* for the serodiagnosis of trichinellosis are superior to the most commonly used classical ML ES antigens, while Ad6+NBL ES antigens are complex to get. Therefore, the Ad3 ES antigens provide a new source of diagnostic antigens. They could be considered as potential early diagnostic antigens for trichinellosis, or the mixture of Ad3 and Ad6+NBL ES might be used. However, the sensitivity and specificity of this method need to be further evaluated in a large-scale study using serum samples infected with *Trichinella* and other helminths.
